# Expression of Stromal Progesterone Receptor and Differential Methylation Patterns in the Endometrium May Correlate with Response to Progesterone Therapy in Endometrial Complex Atypical Hyperplasia

**DOI:** 10.1007/s43032-020-00175-w

**Published:** 2020-03-02

**Authors:** Adam S. Neal, Miguel Nunez, Tiffany Lai, Anela Tosevska, Marco Morselli, Malaika Amneus, Mae Zakhour, Neda A. Moatamed, Matteo Pellegrini, Sanaz Memarzadeh

**Affiliations:** 1 Department of Obstetrics and Gynecology, David Geffen School of Medicine, University of California Los Angeles, Los Angeles, CA 90095 USA; 2 UCLA Eli and Edythe Broad Center of Regenerative Medicine and Stem Cell Research, University of California Los Angeles, Los Angeles, CA 90095 USA; 3 University of California San Fransisco School of Medicine, 516 Parnassus Avenue, San Fransisco, CA 94143 USA; 4 Department of Molecular, Cell and Developmental Biology, University of California Los Angeles, Los Angeles, CA 90095 USA; 5 Institute for Genomics and Proteomics, University of California Los Angeles, Los Angeles, CA 90095 USA; 6 Institute for Quantitative and Computational Biology—The Collaboratory, University of California Los Angeles, Los Angeles, CA 90095 USA; 7 Department of Obstetrics and Gynecology, Southern California Permanente Medical Group, Panorama City, CA 91402 USA; 8 Department of Pathology and Laboratory Medicine, David Geffen School of Medicine, University of California Los Angeles, Los Angeles, CA 90095 USA; 9 Molecular Biology Institute, University of California Los Angeles, Los Angeles, CA 90095 USA; 10 The VA Greater Los Angeles Healthcare System, Los Angeles, CA 90073 USA

**Keywords:** Progesterone therapy, Endometrial hyperplasia, Biomarkers, Progesterone receptor, Hormonal therapy

## Abstract

Progesterone therapy is a viable treatment for complex atypical hyperplasia (CAH) and endometrial adenocarcinoma, though reliable molecular determinants of response are not available. To explore if analysis of pre-therapy endometrial biopsies could yield biomarkers of response to progesterone, patients with CAH or adenocarcinoma undergoing treatment with progestins were included in this cross-sectional study. Immunohistochemistry for progesterone receptor (PR) was performed. Manual PR expression scores (PRES) were first calculated for biopsies by counting PR-positive nuclei in 12 sensitive vs 9 resistant samples. Significant differences in manual PRES were detected in the stroma (*p* < 0.01) and total endometrium (*p* < 0.01) for sensitive vs resistant patients. Manual PRES in the stroma had the highest accuracy in segregating sensitive vs resistant patients (96%). Differences in epithelial PRES were not significant. To validate these findings, a correlation between manual PRES and visual PRES was performed in the 21 patients. An additional 11 patients were analyzed to test if visual PRES would be predictive of response to progesterone. Visual PRES in epithelia and stroma in the 32 specimens was calculated. Significant differences in visual PRES were detected in the stroma for sensitive vs resistant samples (*p* < 0.01), while differences in epithelial and total endometrium were not significant. Whole genome bisulfite sequencing was performed on DNA isolated using pre-therapy biopsies from 6 sensitive and 6 resistant patients in this cohort. Differentially methylated regions were identified in the stroma and epithelium when evaluating sensitive vs resistant samples. Pathways involved in cell adhesion demonstrated the greatest difference in methylation in these samples.

**Electronic supplementary material:**

The online version of this article (10.1007/s43032-020-00175-w) contains supplementary material, which is available to authorized users.

## Introduction

Endometrial cancer is the most common gynecologic cancer in the USA with an estimated 63,000 new cases in 2018[1]. Although endometrial adenocarcinoma is predominantly diagnosed in postmenopausal women, premenopausal women are also diagnosed with this disease. Endometrial hyperplasia, specifically complex atypical hyperplasia (CAH), is a precursor for endometrial cancer [2]. In fact, 42% of women with CAH are diagnosed with concurrent endometrial adenocarcinoma at the time of hysterectomy [3]. Hormonal therapy using progesterone has been shown to be efficacious in the treatment of both conditions [4, 5]. Progestin therapy is considered in women who wish to preserve fertility, are poor surgical candidates, or for patients with widely metastatic primary or recurrent disease. However, there are no reliable molecular determinants of efficacy, making it difficult to predict response to this treatment. The incidence of endometrial adenocarcinoma among young women is on the rise in the USA [1]. This is partly due to the rise in the rate of obesity, a well-known risk factor for both CAH and endometrial adenocarcinoma [6]. As women are delaying childbearing, we predict the use of hormonal therapy for treatment of CAH, and endometrial cancer will become more relevant, making it paramount to determine biomarkers of response.

As the endometrium is a hormonally regulated tissue, many studies have investigated the prognostic significance of both progesterone receptor (PR) and estrogen receptor (ER) in determining response to progesterone [7–10]. The majority of these studies quantified the expression of steroid hormone receptor indiscriminate of stromal or epithelial cellular compartments. As endometrial cancers and hyperplasias are characterized by expansion of epithelial cells, the expression profile in stromal cells is often overlooked. Paracrine signaling between stromal and epithelial cells is critical in müllerian duct development, and steroid hormone receptors are important for mediating these actions [11]. The crosstalk between cancer and stromal cells is important for the development of hormonally regulated neoplasms [12, 13]; thus, analysis of each cellular compartment independently may be relevant.

In addition to expression of hormone receptors that may mediate progesterone therapeutic effects in the stroma or epithelium, independent analysis of the methylome in each cellular compartment may also yield insight into the emergence of hormone therapy resistance. DNA methylation is an epigenetic modification that can cause altered gene expression and malignant cellular transformation. Recent studies have suggested that these epigenetic changes may play a role in cancer initiation or metastasis [14]. As these epigenetic changes can be modulated with pharmacologic agents such as DNA methyltransferase inhibitors or histone deacetylase inhibitors, understanding the epigenetic landscape may provide therapeutic opportunities. Currently, limitations exist in the clinical application of small molecule epigenetic modifiers, particularly in solid tumors [15–17]. The epigenetic profile of tumor epithelial cells and their associated microenvironment are currently being explored in other tumor models such as prostate and breast cancer [18, 19]. Although the epigenetic landscape has been investigated in endometrial cancer [20–24], there is limited analysis independently examining epithelial and stromal compartments.

This study investigates whether stromal or epithelial PR expression correlates with favorable response to progesterone treatment in a cohort of thirty-two patients diagnosed with either CAH or endometrial adenocarcinoma. Additionally, whole genome bisulfite sequencing (WGBS) was used to evaluate the epigenetic profile in the stroma and epithelium in six patients who had a favorable response to progesterone therapy and six patients with progesterone-resistant disease.

## Materials and Methods

### Subjects

Women with a diagnosis of CAH or well-differentiated endometrial adenocarcinoma who had been treated with progestins were identified at two clinical sites through an IRB-approved protocol (IRB# 11-000899). After discussing treatment options, all patients had opted for progesterone therapy. Based on review of medical records, patients had not received prior treatment. Endometrial biopsies were obtained for all patients prior to initiation of progesterone therapy and analyzed for initial diagnosis (pre-therapy biopsy). Follow-up biopsies collected during treatment were histologically examined by a pathologist with expertise in gynecologic malignancies to classify response to progesterone (post-therapy biopsy). Sensitivity to progesterone was defined as CAH or well-differentiated endometrial adenocarcinoma that resolved after hormonal therapy with progesterone. Resolution was defined as negative endometrial sampling post-therapy or negative pathology on final hysterectomy. Resistance was defined as residual CAH or well-differentiated endometrial adenocarcinoma on biopsy or hysterectomy specimen on treatment after at least 6 months of hormonal therapy.

### Inclusion Criteria

Review of all slides by an expert pathologist, Dr. Neda A. Moatamed, was performed to determine inclusion in our study. Inclusion criteria for all identified samples were the following: (1) no evidence of exogenous progesterone effects in pre-therapy slides and (2) confirmation of diagnosis and response or resistance to therapy. An additional inclusion criterion was a sufficient number of FFPE slides from pre-therapy biopsies or final hysterectomy specimens to perform analyses in this study.

Thirty patients diagnosed with CAH or well-differentiated endometrial adenocarcinoma, diagnosed and treated at Olive View Medical Center in Sylmar, CA, were identified by Dr. Malaika Amneus and Dr. Mae Zakhour. Fourteen additional patients were identified at UCLA Medical Center by Dr. Neda A. Moatamed and Dr. Sanaz Memarzadeh. Twelve samples were removed from this study as they did not meet inclusion criteria outlined above. The remaining 32 samples were included in our study. Twenty patients in this cohort were classified as progesterone-sensitive and 12 were classified as having progesterone-resistant disease. Demographic and treatment information for this patient cohort is reported (Table 1).

**Table 1:** Patient demographics

	Resistant (*n* = 12)	Sensitive (*n* = 20)	*p* value
Age (median, mean ± SEM)	32.0, 36.4 ± 3.0	36.5, 36.7 ± 1.5	*p* = 0.43
BMI (median, mean ± SEM)	34.5, 39.8 ± 3.9	31.0, 32.8 ± 2.2	*p* = 0.12
Race			*p* = 0.25
White, non-hispanic	5, 41.7%	4, 20.0%	
White, hispanic	7, 58.3%	8, 40.0%	
Pacific islander	-	1, 5.0%	
Asian	-	5, 25.0%	
Black, non-hispanic	-	1, 5.0%	
Unknown	-	1, 5.0%	
Type of progestin treatment			*p* = 0.38
Medroxyprogesterone acetate	4, 33.3%	7, 35%	
Megestrol acetate	6, 50%	9, 45%	
Levonorgestrel intrauterine system	11, 91.7%	12, 60%	
Progesterone micronized	1, 8.3%	2, 10%	
Norethindrone acetate	-	1, 5%	
Initial diagnosis			*p* = 0.13
Complex atypical hyperplasia	10, 83.3%	20, 100%	
Well-differentiated endometrial cancer	2, 16.7%	-	
Length of treatment (Q1–median–Q3)	8.75–17.5–31	7–9–13.5	*p* = 0.05

### Immunohistochemistry

Immunohistochemistry (IHC) was performed on formalin-fixed paraffin-embedded 5 μm sections from endometrial biopsies. Expression of PR was visualized using anti-PR (LabVision RM-9102-S0, Thermo Fisher Scientific) at 1:250; ER was evaluated using anti-ER-alpha (Abcam ab80922, 1:250), and Ki67 was visualized using anti-Ki67 (Dako, MIB-1, 1:100). Biotinylated goat anti-rabbit or biotinylated rabbit anti-mouse (Jackson ImmunoResearch, 1:1000) followed by streptavidin-conjugated horseradish peroxidase (HRP, Jackson ImmunoResearch, 1:1000) was used for DAB staining.

### Manual Quantification of Progesterone Receptor

To quantify expression of PR in the epithelium and stroma of endometrial specimens, stained sections were scanned, and five random fields of view were visualized at × 20 magnification and quantified using the ImageJ cell counter function. The percent PR-positive cells (total number PR-positive cells/total number nuclei) were quantified manually in the stroma and epithelium for each field of view. PR-positive expression was also quantified in total endometrium. To ensure accuracy of this method, two investigators independently quantified PR expression in five endometrial biopsies using five different fields of view for each biopsy. The percent PR-positive cells quantified by each investigator were consistent for epithelial and stromal compartments for each biopsy, suggesting reproducibility of this measurement. Two independent investigators calculated the intensity or H-score on a scale of 0–3; 0 = no staining, 1 = weak, 2 = moderate, and 3 = strong (Supplementary Fig. 1a). Correlation between H-scoring by the two investigators demonstrated concordance of staining intensity assessment **(**Supplementary Fig. 1b). The percentage of PR-positive nuclei was multiplied by the H-score [25–27] for each field of view, and the average percentage of the five fields of view is reported as the manual PR expression score (PRES).

### Visual Quantification of Progesterone and Estrogen Receptor

In order to identify a more clinically feasible method of quantifying hormone receptor expression, PR or ER expression was evaluated by study pathologist, Dr. Neda A. Moatamed, following IHC staining. Briefly, each IHC-stained biopsy slide was visually inspected, and the percentage of hormone receptor positive cells was estimated in the epithelia, stroma, and in total endometrium along with an H-score ranging from 0 to 3; 0 = no staining, 1 = weak, 2 = moderate, and 3 = strong. For this analysis, hormone receptor percentages and intensities were estimated focusing on regions of tissue that exhibited hyperplastic characteristics, verified using adjacent H&E slides. Finally, the percentage of hormone receptor positive cells was multiplied by the H-score to yield either a visual PR expression score (PRES) or ER expression score (ERES).

### Statistical Analysis

Non-parametric receiver operator characteristic (ROC) analysis was used to find a PRES threshold and compute accuracy statistics to determine PR expression in which cellular compartment best predicts response to hormonal treatment. Accuracy is defined as the average of the percent correctly classified sensitive and percent correctly classified resistant. The *p* values for comparing proportions were computed via Fisher’s exact test. The *p* values for mean comparisons with normally distributed data were computed via *t* tests, and the *p* values for median comparisons including PR expression medians and ER expression medians were compared via the non-parametric Wilcoxon rank sum test. The association between manual PRES vs visual PRES, or PRES vs ERES was quantified using the Spearman rank correlation (r_s_).

### Laser Capture Microdissection

Formalin-fixed paraffin-embedded 5 μm sections were deparaffinized by washing in xylenes. Sections were rehydrated, stained with hematoxylin, and then rinsed in bicarbonate. Zeiss PALM CombiSystem laser capture microdissection (LCM) microscope was used to cut at least a 100,000 μm^2^ area for each pre-therapy biopsy. DNA was extracted using a commercially available kit designed for use with paraffin-embedded tissues (Qiagen).

### Preparation of DNA, Whole Genome Bisulfite Sequencing, and Analysis of Data

Whole genome bisulfite sequencing was performed on stroma and epithelia from 7 sensitive and 7 resistant patients. DNA extracted from LCM-isolated cells was treated with bisulfite using the Zymo Lightning Conversion Reagent (Zymo Research) followed by desulfonation following the recommended protocol. The libraries were prepared using the Pico-Methyl kit (Zymo Research) according to manufacturer’s instructions. Final libraries were purified using 1x SPRI beads (Beckman Coulter), visualized on an Agilent TapeStation D1000 (Agilent Technologies), and quantified using a Qubit HS assay (Thermo Fisher Scientific). Once sequenced, filtered fastq files were trimmed using cutadapt version 2.4 [28]. Alignment of raw reads to the hg38 reference genome was performed using BSBolt (https://bsbolt.readthedocs.io/en/latest/), and PCR duplicates were removed using samtool markup (http://www.htslib.org/). Two patient samples were excluded from downstream analysis due to low sequencing coverage and methylation call bias with high levels of non-CpG methylation. Methylation calling was performed on pooled biological replicates using cgmaptools [29]. We chose to pool samples as the sequencing coverage was less than 10x which resulted in the loss of many CpGs. CpG sites with at least 10x coverage across all pools were selected for downstream analysis, yielding a total number of around 4 million sites. Differentially methylated regions (DMRs) were identified using metilene v0.2–8 [30] using the Mann-Whitney *U* test and two-dimensional Kolmogorov-Smirnov statistical testing, with a minimum DNA methylation change set to 0.3. DMR lists were subsequently annotated using GREAT version 4.0.4 [31] against the whole genome as background, with an association set to 5 kb upstream and 1 kb downstream from the transcriptional start site (TSS) for proximal regions and 5 kb for distal regions. Gene ontology (GO) and pathway enrichment analysis were performed on all identified DMRs using metascape [32].

## Results

### Levels of Stromal, but Not Epithelial, Progesterone Receptor Expression Correlated with Favorable Response to Progestin Treatment in Patients with Complex Atypical Hyperplasia/Well-Differentiated Endometrial Adenocarcinoma

Standard treatment for CAH or endometrial adenocarcinoma involves definitive surgery with hysterectomy and bilateral salpingo-oophorectomy. However, in a subset of patients who want to pursue childbearing or are poor surgical candidates, hormonal therapy may be an option. Response rates for hormonal therapy alone vary between 76 and 85% in patients with endometrial hyperplasia or well-differentiated adenocarcinoma [5, 33]. Currently, there are no reliable means to accurately predict response.

**Fig. 1:**
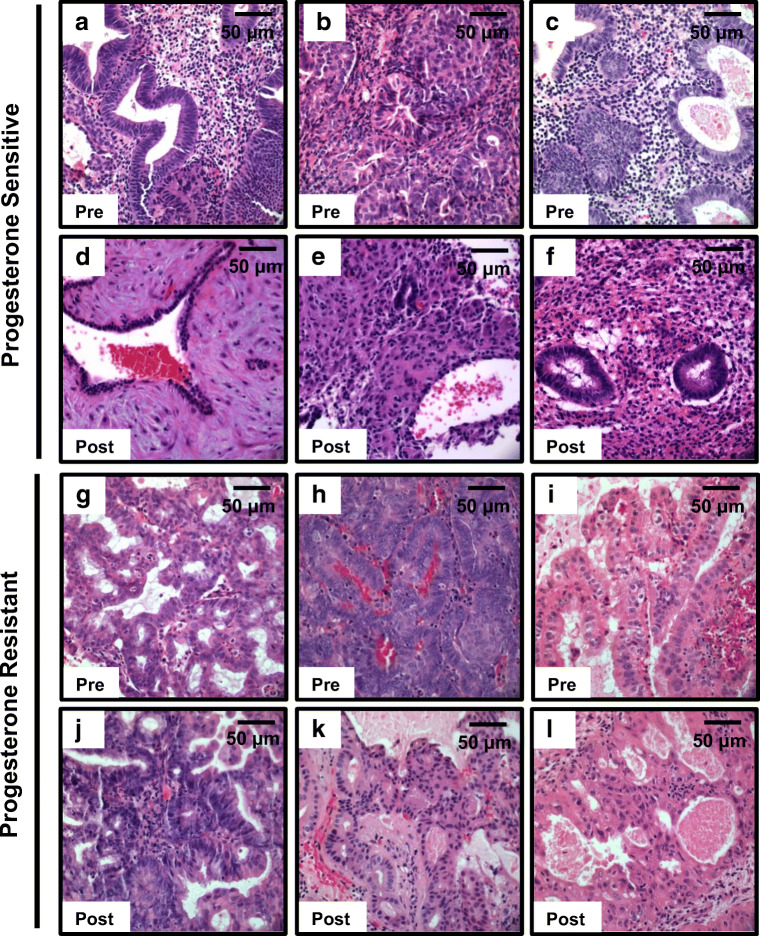
Pre-therapy and post-therapy hematoxylin and eosin endometrial biopsy sections obtained from progesterone sensitive and resistant patients. All patients underwent endometrial biopsies prior to initiating treatment for initial diagnosis (pre-therapy). The same patients underwent an additional endometrial biopsy or hysterectomy during therapy to assess for progesterone efficacy (post-therapy). Pre-therapy histologic sections from patients sensitive to progesterone (**a**–**c**). Patient-matched post-therapy histologic sections showed extensive hormonal effects at the end of treatment (**d**–**f**). Pre-therapy histologic sections from patients resistant to progesterone (**g**–**i**). Patient-matched post-therapy histologic sections showed persistent complex atypical hyperplasia or hyperplasia without atypia (**j**–**l**)

Pre-therapy endometrial biopsies for the subjects included in this study **(**Table 1**)** were obtained prior to initiation of treatment. Tissue from follow-up biopsies or hysterectomy was collected during treatment (post-therapy biopsy) **(**Fig. 1**)**. The majority of patients (28/32) were diagnosed with CAH; 2/32 patients were diagnosed with CAH approaching endometrial cancer, and 2/32 patients were diagnosed with well-differentiated adenocarcinoma. Ki67 expression was estimated in both pre- and post-therapy biopsies and compared in sensitive vs resistant samples (*n* = 3 sensitive and *n* = 3 resistant, Supplementary Fig. 2a). Results demonstrate a decrement in Ki67 expression after administration of progesterone hormonal therapy in both sensitive and resistant groups (Supplementary Fig. 2b).

**Fig. 2:**
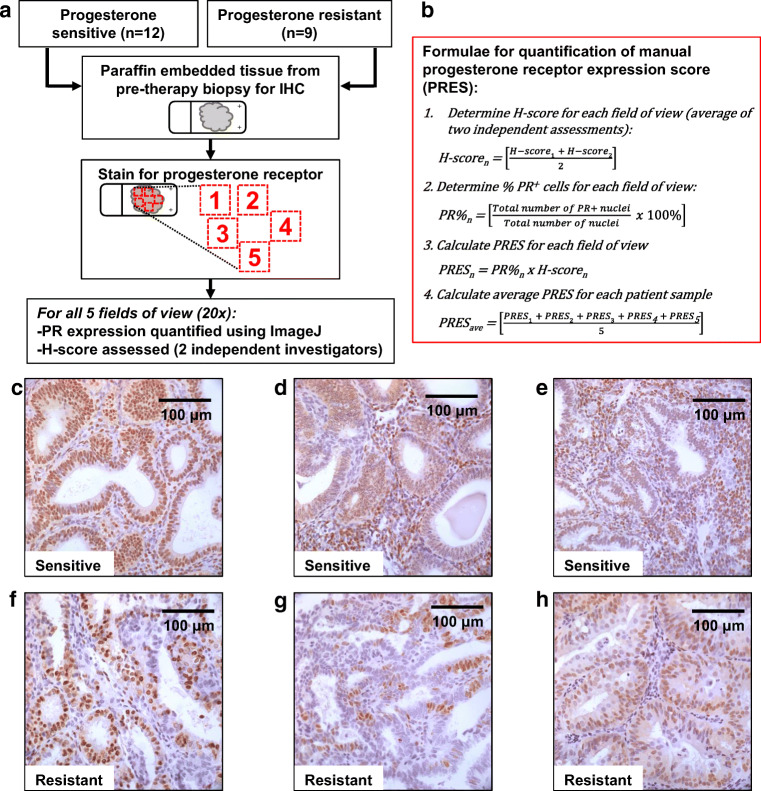
Quantification of manual progesterone receptor expression score in pre-therapy endometrial biopsies. **a** Schema: 12 progesterone-sensitive and 9 progesterone-resistant patients were included in this analysis. Pre-therapy endometrial biopsies were stained for progesterone receptor (PR). PR expression was quantified in the epithelium and stroma via ImageJ in 5 independent fields of view. Total PR expression was obtained by adding the number of PR-positive nuclei in the epithelium and stroma. H-score was determined by 2 independent investigators. **b** PR expression scores (PRES) were calculated using the formulae shown. Representative images of PR expression are shown in 6 independent patient samples, 3 sensitive **(c**–**e)**, and 3 resistant **(f**–**h)** to hormonal therapy

**Fig. 3:**
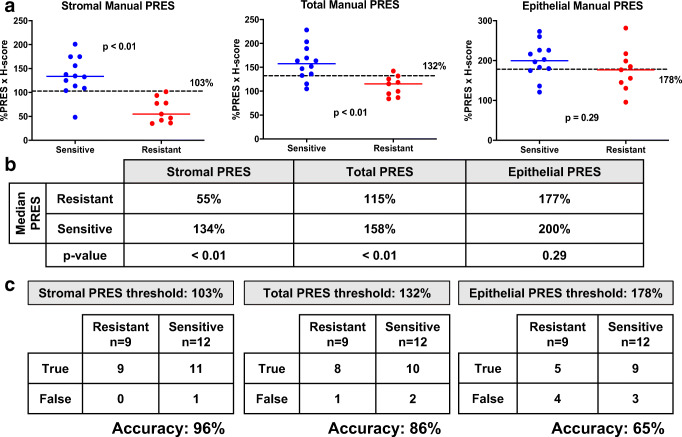
Stromal, but not epithelial, manual progesterone receptor expression score (PRES) correlates with favorable response to progestin therapy. Manual PRES were calculated for each cellular compartment (stromal, epithelial, total endometrial cells) and compared between 12 sensitive and 9 resistant samples. **a** There was a statistically significant difference in stromal manual PRES between the sensitive vs resistant samples (*p* < 0.01). The difference in total manual PRES between the sensitive and resistant groups was also statistically significant (*p* < 0.01). Significant differences in epithelial manual PRES between progesterone-sensitive vs -resistant samples were not detected. **b** Median manual PRES and *p* values comparing sensitive vs resistant samples for each cellular compartment (stromal, epithelial, total). **c** Confusion matrices for stromal, epithelial, and total manual PRES reporting the statistics which were used to determine accuracy of biomarker. Accuracy was calculated by obtaining the mean % correct sensitive and % correct resistant. A stromal manual PRES threshold of 103% had the highest accuracy in predicting response to treatment (96%), followed by a total manual PRES threshold of 132% (86% accuracy). An epithelial manual PRES threshold of 177% had the lowest accuracy in predicting response to treatment (65%). True is defined as resistant samples with manual PRES values below the threshold, and sensitive samples with manual PRES values above the threshold

Manual PRES was examined in both epithelial and stromal compartments in pre-therapy endometrial biopsies for 12 sensitive and 9 resistant patient samples using IHC (Fig. 2). Manual PRES in these pre-therapy biopsies was determined by multiplying percentage of PR-positive nuclei by H-score for each subject. This calculated score was then used to compare sensitive vs resistant groups **(**Fig. 3**)**.

Manual PRES for each of the endometrial cellular compartments (stroma or epithelium) was independently analyzed. A similar analysis was completed for total (stroma + epithelium) endometrial cells (total manual PRES). Stromal and total manual PRES were significantly higher in biopsies from patients sensitive to progesterone treatment compared with resistant (Fig. 3a). In contrast, epithelial manual PRES was not significantly different in sensitive vs resistant samples (Fig. 3a). The median manual PRES in the stroma for sensitive patients was 134% compared with 55% in the resistant group (*p* < 0.01, Fig. 3b). The median total manual PRES in the sensitive vs resistant groups was also statistically significant (158% vs 115% respectively, *p* < 0.01, Fig. 3b). There was no difference in the epithelial manual PRES between the sensitive and resistant groups (200% vs 177% respectively, *p* = 0.29, Fig. 3b). ROC analysis was used to determine a threshold at which the response to treatment prediction accuracy was highest. The stromal PRES threshold of 103% was able to predict 9/9 resistant and 11/12 sensitive biopsies (96% accuracy, Fig. 3c). In comparison, a total PRES threshold of 132% could predict 8/9 resistant and 10/12 sensitive biopsies (86% accuracy, Fig. 3c). Finally, an epithelial PRES threshold of 178% was only 65% accurate, predicting 5/9 resistant and 9/12 sensitive samples (Fig. 3c).

**Fig. 4:**
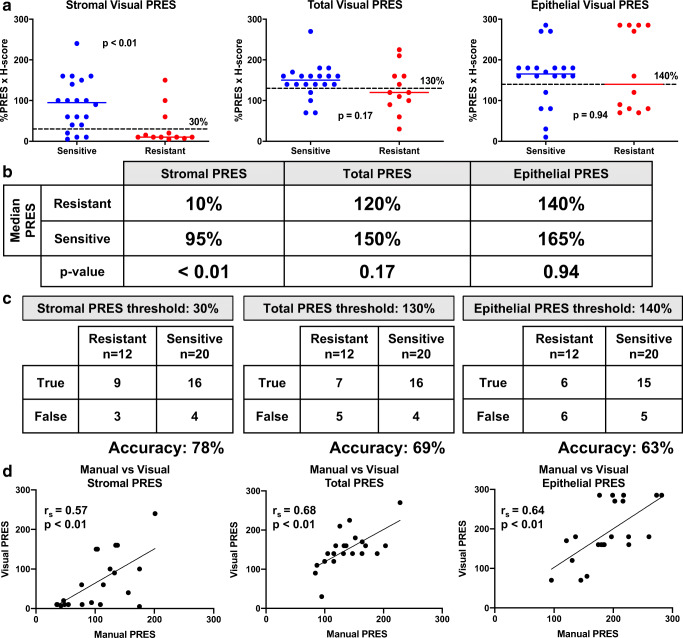
Visual progesterone receptor expression scoring (PRES) confirmed stromal PR expression may correlate with progesterone hormonal sensitivity. Visual PRES was calculated for each cellular compartment (stromal, epithelial, total endometrial cells) and compared between sensitive and resistant samples. **a** Visual PRES in stromal cells was significantly lower in samples resistant to progesterone therapy compared with sensitive (*p* < 0.01). Differences in epithelia and in total endometrial cells were not significant (*p* = 0.94 and *p* = 0.17, respectively). **b** Median visual PRES and *p* values comparing sensitive vs resistant samples for each cellular compartment (stromal, epithelial, total). **c** Confusion matrices for stromal, epithelial, and total visual PRES. A stromal visual PRES threshold of 30% had the highest accuracy in predicting response to treatment (78%), followed by a total visual PRES threshold of 130% (69% accurate). An epithelial visual PRES threshold of 140% had the lowest accuracy in predicting response to treatment (63% accurate). True is defined as resistant samples with visual PRES values below the threshold, and sensitive samples with visual PRES values above the threshold. **d** Correlation between manual PRES and visual PRES for all pre-therapy biopsies quantified in the stroma, epithelia, and in total tissue. Spearman correlation coefficients (*r*_s_) calculated for stroma (*r*_s_ = 0.57), epithelial (*r*_s_ = 0.68), and in total endometrial cells (*r*_s_ = 0.64) demonstrate a moderate positive correlation

In order to validate these findings and expand our cohort of patient samples, visual PRES was calculated for the 21 patients counted manually and an additional 11 pre-therapy biopsies (*n* = 32 total). Again, stromal visual PRES was significantly higher in biopsies from patients sensitive to progesterone treatment compared with resistant (Fig. 4a). Here, no statistically significant difference was observed in either epithelial or total visual PRES (Fig. 4a). The median visual PRES in the stroma for sensitive patients was 95% compared with 10% in the resistant group (*p* < 0.01, Fig. 4b). There was no difference in the median epithelial or total visual PRES between the sensitive and resistant groups (*p* = 0.94 and *p* = 0.17, respectively, Fig. 4b). A similar ROC analysis as reported for manual PRES was employed. The stromal visual PRES threshold of 30% was able to predict 9/12 resistant and 16/20 sensitive biopsies (78% accuracy, Fig. 4c). In comparison, epithelial and total visual PRES were only 63% and 69% accurate in predicting response to therapy, respectively (Fig. 4c). Comparison of manual and visual PRES methodologies for each sample tested is reported, demonstrating a positive but not perfect correlation (Fig. 4d). Spearman correlation coefficients (*r*_s_) for stroma, epithelia, and in total tissue range from 0.57 to 0.68 indicating a moderate positive correlation. Expression of PR in post-therapy biopsies is shown in both sensitive and resistant samples (Supplementary Fig. 3).

**Fig. 5:**
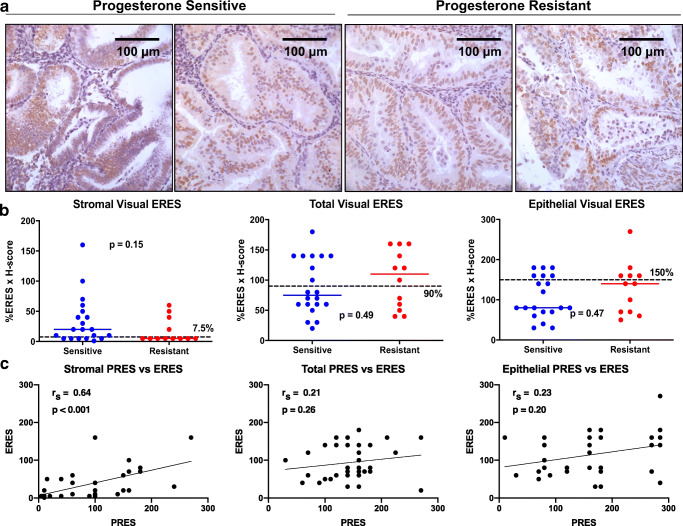
Expression of estrogen receptor did not correlate with response to progesterone. **a** Representative images of estrogen receptor (ER) stained pre-therapy endometrial biopsies from two patients sensitive to progesterone and two patients resistant to treatment in our cohort. **b** ER was quantified using the visual quantification method to yield a visual ER expression score (visual ERES) and compared in sensitive vs resistant samples. Median visual ERES was not different in sensitive vs resistant samples for endometrial stroma (*p* = 0.15), epithelia (*p* = 0.47), or in total tissue (*p* = 0.49). **c** Scatterplots illustrating correlation between visual PRES and visual ERES measured in our analysis revealed that stromal PR and ER expression are moderately correlated in the stroma (*r*_s_ = 0.64, *p* < 0.001). PR and ER expression in epithelia (*r*_s_ = 0.23, *p* = 0.20) and in total endometrial tissue (*r*_s_ = 0.21, *p* = 0.26) demonstrated a weak correlation

Next, estrogen receptor (ER) was quantified using IHC in the same patient samples using the visual methodology (Fig. 5a). ER expression was compared in progesterone-sensitive vs -resistant samples (Fig. 5b). In this analysis, median ERES was not statistically different in sensitive vs resistant samples in either the stroma (*p* = 0.15), epithelia (*p* = 0.47), or in total endometrial tissue (*p* = 0.49) (Fig. 5b). A comparison between visual PRES and visual ERES revealed that stromal PR and ER expression had a moderate positive correlation (*r*_s_ = 0.64, *p* < 0.001, Fig. 5c); whereas PR and ER expression in the epithelia and in total endometrial tissue demonstrated only a weak correlation (Fig. 5c).

The significance of PR expression in the stroma of this cohort correlates with preclinical studies that have suggested progesterone anti-tumor effects may be mediated through the stroma [34, 35]. Endometrial stromal expression of HAND2, a transcription factor regulated by PR, has been implicated in the antiproliferative actions of progesterone in the endometrium by repressing fibroblast growth factor expression during pregnancy [34]. Additionally, methylation of HAND2 has been observed in atypical hyperplasia, suggesting that silencing of this gene may be an early step in carcinogenesis [36]. Analysis of this patient cohort suggests that stromal PR may play an important role in mediating sensitivity to progestin therapy as stromal PRES correlates well with response to progesterone. Additionally, this study suggests that stromal PR expression may be a potential biomarker of response to progesterone therapy. Further large prospective studies would need to validate this observation.

### Differentially Methylated Regions Present in the Epithelium and Stroma of Progesterone-Resistant Vs Progesterone-Sensitive Endometrium

Epigenetic changes have been studied extensively in many cancers. Alterations in DNA methylation have been implicated in carcinogenesis, and due to its stability, DNA methylation is an attractive biomarker of disease. For example, methylation of SHOX2 is being used to explore differences between benign and malignant lung tissue [37]. Additionally, SEPT9 methylation is being examined as a potential screening tool for colorectal cancer [38]. DNA methylation has also been identified as a prognostic marker: epigenetic silencing of 0(6)-methylguanine-DNA-methyltransferase in glioblastoma has been correlated with increased progression free survival [39]. From our cohort of thirty two patients, a subset of progesterone-sensitive and -resistant samples was evaluated by WGBS to explore potential DMRs in the epithelium and stroma.

**Fig. 6:**
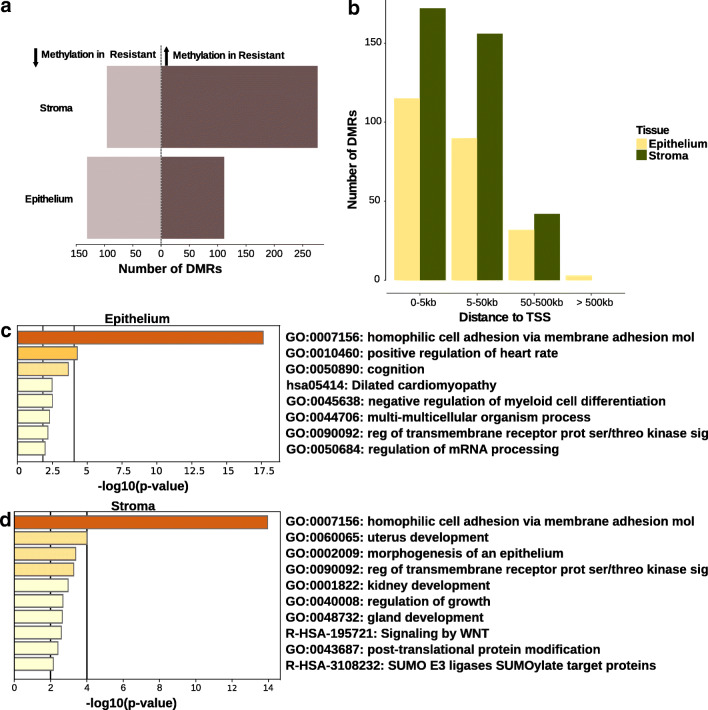
Differentially methylated regions in the stroma and epithelium of resistant vs sensitive patients. **a** The number of differentially methylated regions (DMRs) with at least 30% difference in DNA methylation in resistant vs sensitive patients. The number of regions with decreased methylation in resistant samples is depicted to the left side of the dotted line (light brown), the regions with increased methylation to the right (dark brown). **b** Number of DMRs per tissue and their proximity to gene transcription start sites (TSS). **c**–**d** Gene ontology and pathway enrichment analysis for genes associated with DMRs (proximal to TSS, 5 kb upstream and downstream of the TSS) in epithelium (**c**) and stroma (**d**). The negative log of the enrichment *p* values are depicted on the x-axes; vertical lines mark arbitrary cut-off *p* values at *p* = 0.01 and *p* = 0.0001. Associated GO terms are depicted on the y-axes

WGBS was carried out in a total of 14 patient samples selected from the PR quantification cohort (7 sensitive and 7 resistant to progesterone). Two samples were removed from analysis due to low sequencing coverage and methylation call bias. DMRs were independently identified in stroma (370 DMRs) and epithelium (240 DMRs) of 6 resistant vs 6 sensitive patients (Fig. 6a). To determine the functional relevance of DMRs found in the stroma and epithelium, we determined their location relative to the nearest transcription start site (TSS). The majority of DMRs in both the stroma and epithelia were located proximal to a TSS suggesting they may play a role in gene regulation (Fig. 6b). GO and pathway enrichment analysis for genes associated with DMRs in the epithelium and stroma were carried out revealing an enrichment for cell adhesion genes (Fig. 6 c and d). The full set of DMRs can be found in the supplementary data file.

## Discussion

Hormone receptor expression in endocrine-related malignancies such as breast cancer has been shown to be an important biomarker of response to treatment [40]. For example, ER-positive breast cancers, as determined by ER expression in the tumor, are treated with tamoxifen, a selective ER modulator that blocks the effects of estrogen-induced proliferation [41]. Additionally, PR status is being explored as a potential biomarker of response to progestin-based therapy in endometriosis [42]. However, the significance of hormone receptor expression for progestin therapy in patients diagnosed with endometrial hyperplasia or adenocarcinoma is inconclusive. Some studies have reported no association between PR expression and response to treatment [7, 43], while others have suggested that higher PR expression is associated with a more favorable response [44].

The current study focused on investigating PR expression independently in the stromal and epithelial compartments of endometrial tissue from a cohort of patients diagnosed with CAH or adenocarcinoma. These patients were treated with progestins, and follow-up histology was obtained by repeat endometrial biopsy or hysterectomy to determine response to therapy. Stromal PRES measured manually and visually was most predictive of response to progesterone therapy in this study. In contrast, in this cohort of patients, epithelial PRES quantification using either method was not predictive of response to progesterone hormonal therapy. Manual and visual PRES measurements did have a moderate positive correlation. Differences in quantification using these two methods are likely attributable to a focus on hyperplastic regions of tissue in the visual PRES method, while manual PRES was quantified using 5 random fields of view imaged at × 20 magnification which could include areas of normal endometrial tissue in addition to hyperplastic regions. While stromal PRES was accurate in predicting response to hormonal therapy in our patient cohort, expression of ER was not significantly different between sensitive vs resistant patients. Taken together, these data suggest that stromal PRES may be a potential molecular determinant of response to treatment. However, we recognize that this is a small patient cohort; therefore, further large-scale studies are necessary to validate the diagnostic value of these findings.

Recent studies have suggested aberrant DNA methylation in endometrial carcinogenesis. Furthermore, epigenetic alterations are distinct in type I and type II endometrial cancers suggesting methylation may play a role in the tumorigenesis of these malignancies [21]. Utilizing LCM of endometrial stromal and epithelial cells from paraffin-embedded tissue followed by WGBS, we compared DMRs in a small cohort of patients (*n* = 6 sensitive, *n* = 6 resistant to progesterone). A pooled analysis demonstrated obvious differences in the methylation pattern of sensitive vs resistant samples. While this analysis demonstrates the feasibility of this approach, limitations in methodology include the use of FFPE clinical material and low sequencing coverage. With these limitations in mind, future consideration can be given to perform such analyses using freshly isolated stroma vs epithelia from dissociated endometrial tissue.

Findings presented here suggest that independent investigation of both epithelial and stromal compartments may be necessary in defining biomarkers of response to hormonal therapy in CAH and endometrial adenocarcinoma. Preliminary results presented here suggest that the expression profile of stromal PR and the epigenetic landscape in both stroma and epithelium differ between patients responsive to progesterone and those resistant to treatment. These observations will need to be validated in future larger scale studies.

## Electronic Supplementary Material

ESM 1 (XLSX 43.2 kb)

ESM 2 (PDF 11.3 mb)

## Abbreviations

CAH: Complex atypical hyperplasia
DAB: 3,3′-Diaminobenzidine
DMR: Differentially methylated region
DNA: Deoxyribonucleic acid
ER: Estrogen receptor
ERES: Estrogen receptor expression score
FFPE: Formalin-fixed paraffin-embedded
GREAT: Genomic regions of enrichment annotations tool
GO: Gene ontology
H&E: Hematoxylin and eosin histologic staining
HRP: Horseradish peroxidase
H-score: Histo score, qualitative measure of staining intensity
IRB: Institutional review board
LCM: Laser capture microscopy
PR: Progesterone receptor
PRES: Progesterone receptor expression score
ROC: Receiver operating characteristic
SPRI: solid phase reversible immobilization
TSS: Transcription start site
UCLA: University of California Los Angeles
μm: Micrometer
WGBS: Whole genome bisulfite sequencing
