# Supplementary material for: Expression of Stromal Progesterone Receptor and Differential Methylation Patterns in the Endometrium May Correlate with Response to Progesterone Therapy in Endometrial Complex Atypical Hyperplasia
Source: Reprod Sci. 2020 Mar 2;27(9):1778–90. doi: 10.1007/s43032-020-00175-w (PMC7395059; doi:10.1007/s43032-020-00175-w)
Supplement: Supplementary file 2 — (PDF 11.3 mb) [file 43032_2020_175_MOESM2_ESM.pdf]

**Supplementary Figure 1. Calculation of H-score for manual progesterone receptor expression scoring**

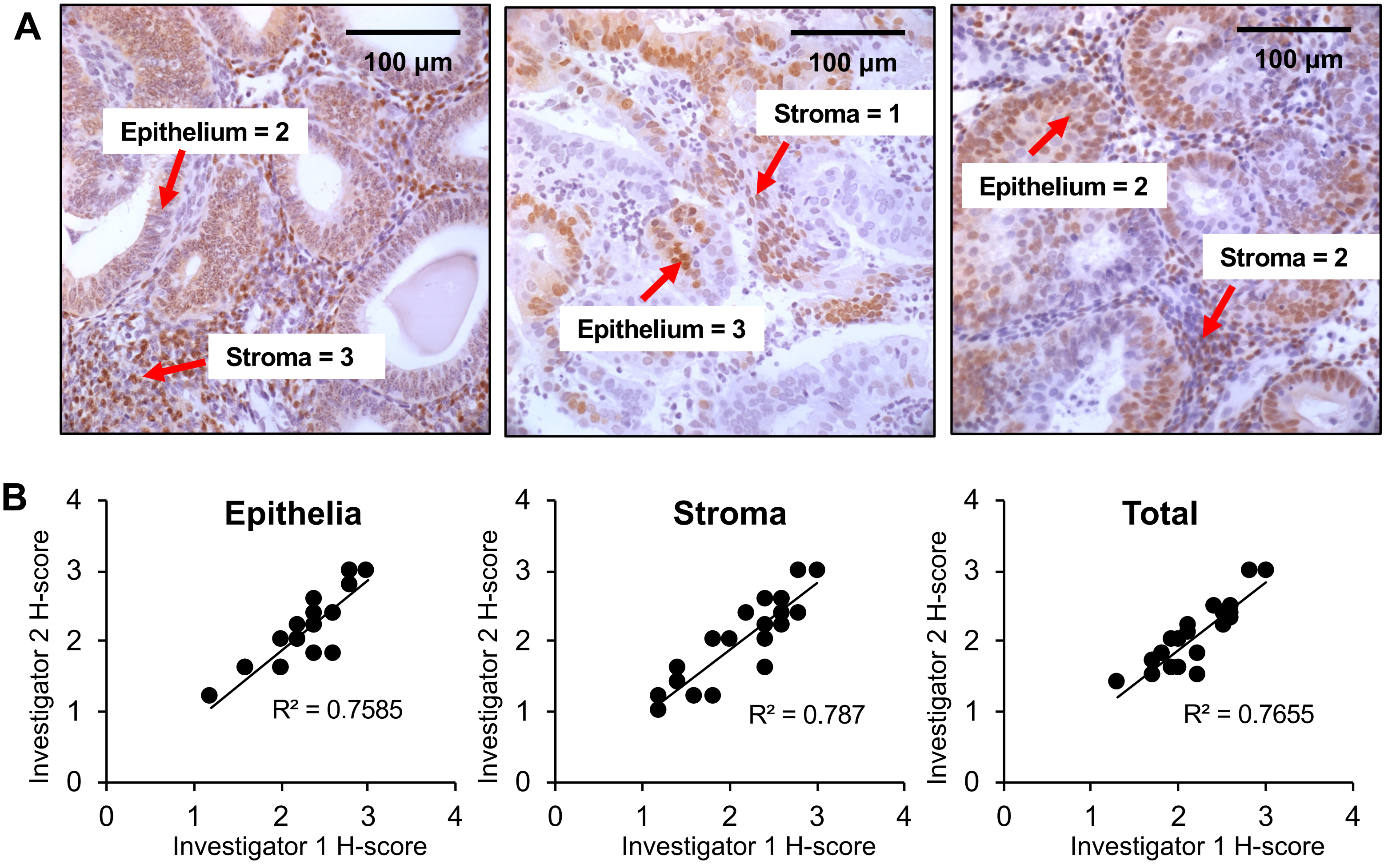


**Supplementary Figure 1. Calculation of H-score for manual progesterone receptor expression scoring. (A)** Stromal and epithelial H-scores for progesterone receptor (PR) expression were determined using a qualitative scoring system (0-3) determined by 2 independent investigators in 12 sensitive and 9 resistant samples. Independent H-score values for each patient sample were averaged for the epithelium and stroma separately. A score of 3=intense/dark staining of nuclei, 2=average staining of nuclei, 1=light staining of nuclei, 0=no staining of PR. Total endometrial H-scores were calculated by averaging the stromal and epithelial scores for each sample. **(B)** Correlation between two investigator reported H-scoring for PR in epithelial, stromal, and in total endometrial tissue shown.

**Supplementary Figure 2. Representative images for Ki67 in pre- and post-therapy endometrial biopsies**

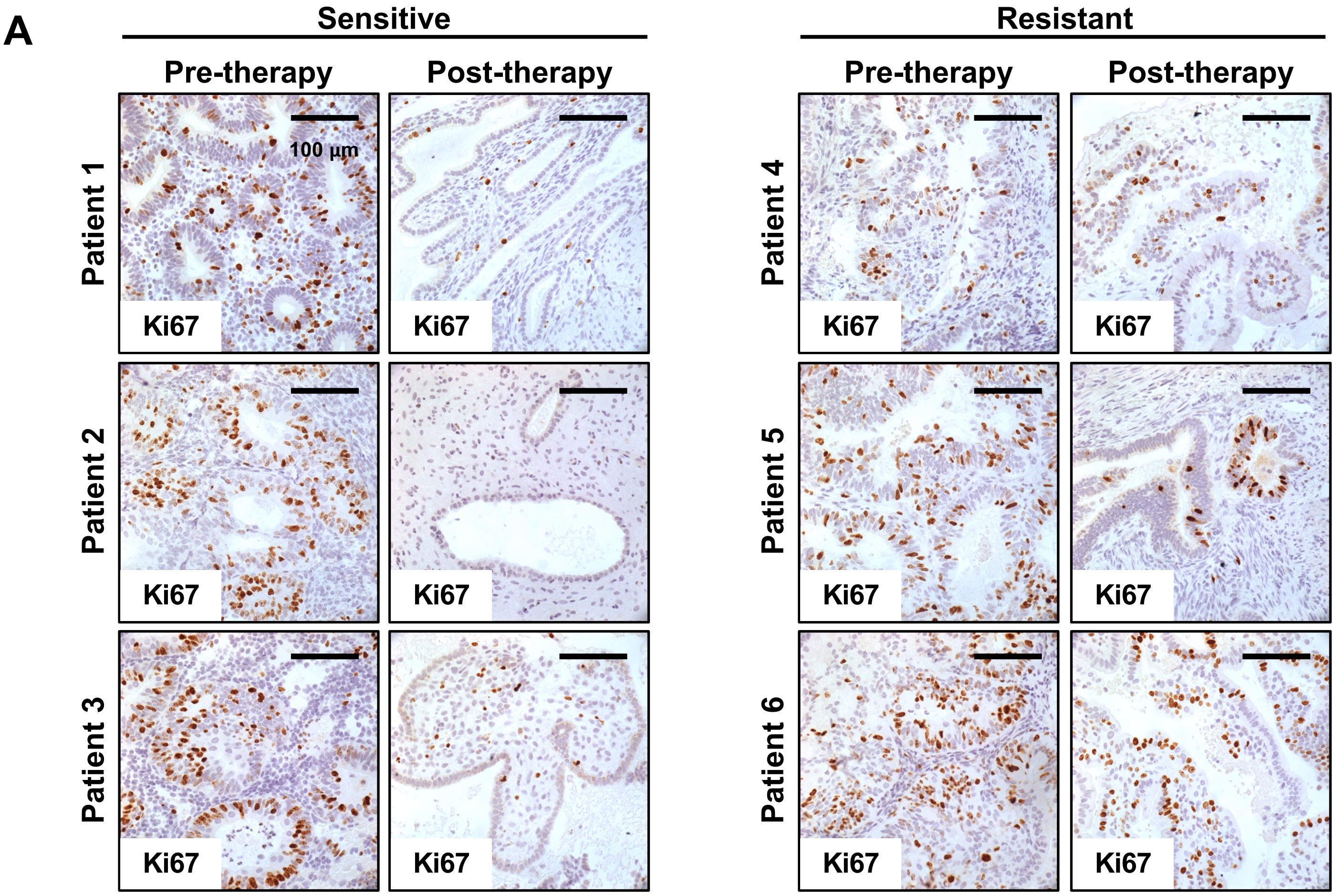


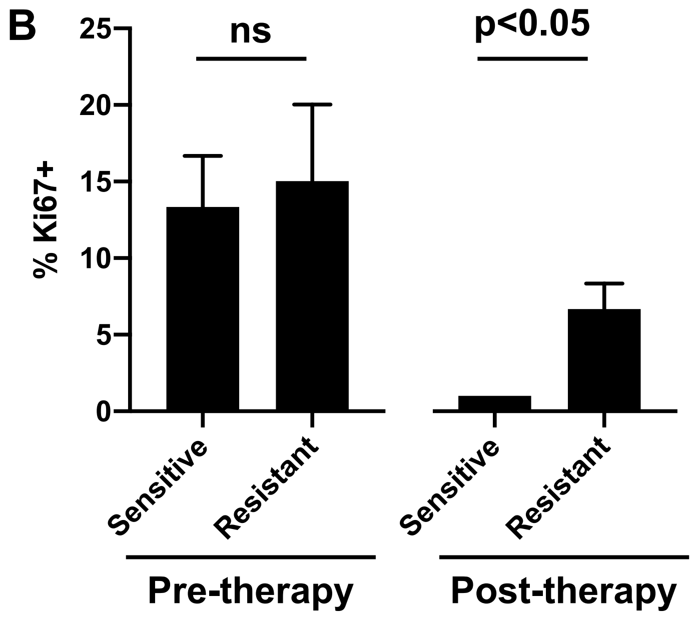


**Supplementary Figure 2. Expression of Ki67 in pre- and post-therapy endometrial biopsies (A)** Representative images of matched pre- and post-therapy endometrial biopsies stained for Ki67. Data shown for three patients sensitive to progesterone therapy and three patients resistant to treatment. **(B)** Quantification of Ki67 in endometrial glands in sensitive and resistant samples (n=3 each). Data demonstrate similar levels of Ki67 expression in endometrial glands in both sensitive and resistant pre-therapy samples with a diminution of Ki67 in post- compared to pre-therapy samples.

**Supplementary Figure 3. Expression of PR after administration of progesterone**

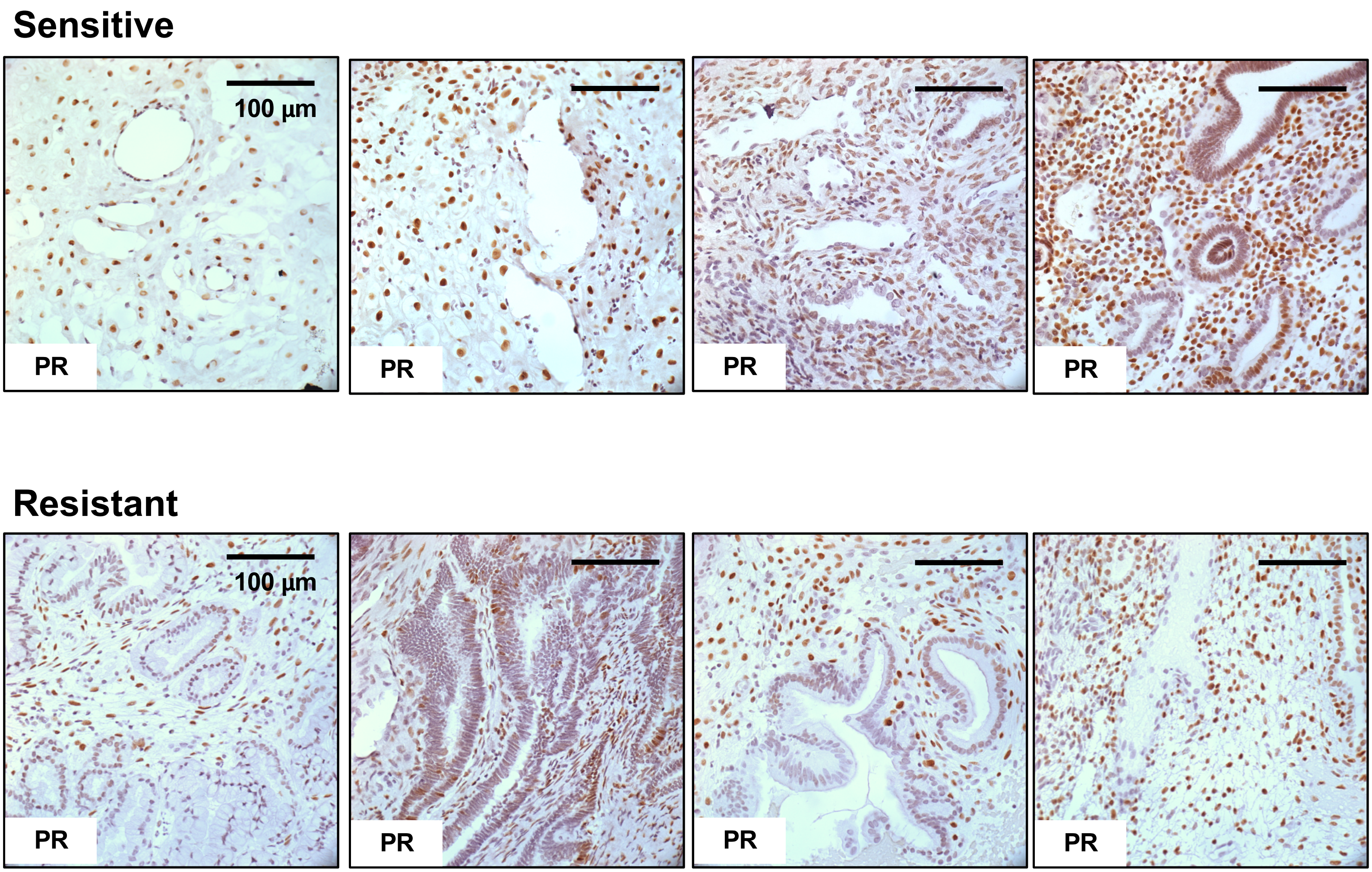


**Supplementary Figure 3. Expression of PR after administration of progesterone.** Representative images of PR staining in post-therapy biopsies obtained after administration of progesterone hormonal therapy.
